# Suggested Involvement of PP1/PP2A Activity and *De Novo* Gene Expression in Anhydrobiotic Survival in a Tardigrade, *Hypsibius dujardini*, by Chemical Genetic Approach

**DOI:** 10.1371/journal.pone.0144803

**Published:** 2015-12-21

**Authors:** Koyuki Kondo, Takeo Kubo, Takekazu Kunieda

**Affiliations:** Department of Biological Sciences, Graduate School of Science, The University of Tokyo, Hongo 7-3-1, Bunkyo-ku, Tokyo 113–0033, Japan; Louisiana State University Health Sciences Center, UNITED STATES

## Abstract

Upon desiccation, some tardigrades enter an ametabolic dehydrated state called anhydrobiosis and can survive a desiccated environment in this state. For successful transition to anhydrobiosis, some anhydrobiotic tardigrades require pre-incubation under high humidity conditions, a process called preconditioning, prior to exposure to severe desiccation. Although tardigrades are thought to prepare for transition to anhydrobiosis during preconditioning, the molecular mechanisms governing such processes remain unknown. In this study, we used chemical genetic approaches to elucidate the regulatory mechanisms of anhydrobiosis in the anhydrobiotic tardigrade, *Hypsibius dujardini*. We first demonstrated that inhibition of transcription or translation drastically impaired anhydrobiotic survival, suggesting that *de novo* gene expression is required for successful transition to anhydrobiosis in this tardigrade. We then screened 81 chemicals and identified 5 chemicals that significantly impaired anhydrobiotic survival after severe desiccation, in contrast to little or no effect on survival after high humidity exposure only. In particular, cantharidic acid, a selective inhibitor of protein phosphatase (PP) 1 and PP2A, exhibited the most profound inhibitory effects. Another PP1/PP2A inhibitor, okadaic acid, also significantly and specifically impaired anhydrobiotic survival, suggesting that PP1/PP2A activity plays an important role for anhydrobiosis in this species. This is, to our knowledge, the first report of the required activities of signaling molecules for desiccation tolerance in tardigrades. The identified inhibitory chemicals could provide novel clues to elucidate the regulatory mechanisms underlying anhydrobiosis in tardigrades.

## Introduction

For terrestrial organisms, desiccation is one of the most commonly encountered environmental stresses. To avoid deleterious water loss, most animals escape from a desiccated environment using their mobility, and retain their body water by the proper intake of water and by preventing surface water evaporation [1,2]. In contrast, some small animals, whose mobility is limited and whose large surface/volume ratio enhances evaporation, have adapted to tolerate a loss of body water in order to withstand a desiccated environment [3]. When encountering desiccation, these animals lose water and enter a metabolically inactive dehydrated state referred to as anhydrobiosis, and resume their metabolic activity upon rehydration.

Tardigrades are tiny animals comprising the phylum Tardigrada, in which more than 1000 species have been reported [4]. All tardigrades are principally aquatic and require surrounding water to grow and reproduce, though some species have anhydrobiotic abilities. When desiccated, anhydrobiotic tardigrades contract their bodies longitudinally with the loss of body water, to form a compact shape called a tun, and are able to tolerate almost complete dehydration [5].

For successful transition to anhydrobiosis, many anhydrobiotic animals require pre-exposure to high humidity conditions, called preconditioning, prior to severe dehydration [6–9]. During preconditioning, animals are thought to sense environmental desiccation and prepare for upcoming severe dehydration. Some anhydrobiotic animals, such as the sleeping chironomid, *Polypedilum vanderplanki*, and the anhydrobiotic nematode, *Aphelenchus avenae*, accumulate huge amounts of a non-reducing disaccharide, trehalose, reaching almost 10% to 18% of the dry weight during the preconditioning period [10,11]. The accumulated trehalose is thought to contribute to protect biomolecules from dehydration stress [12]. In the anhydrobiotic tardigrade *Richtersius coronifer*, trehalose is accumulated up to 2.3% of the dry weight upon desiccation, though the accumulated amounts in tardigrades are generally much lower than those in anhydrobiotic arthropods or nematodes [13]. Additionally, energy deprivation by treatment with a mitochondrial uncoupler severely impairs the anhydrobiotic survival of *R*. *coronifer*, suggesting the presence of energetically controlled processes during the transition to anhydrobiosis [14]. To date, most studies of the molecular mechanisms of anhydrobiosis in animals have focused mainly on protective molecules, such as trehalose [12,13], molecular chaperones [15,16], and putative protective intrinsically disordered proteins, including late embryogenesis abundant proteins [17,18] and abundant heat-soluble proteins unique to tardigrades [19,20]. In contrast, the regulatory mechanisms governing the production of such protective molecules and inducing the orchestrated transformation to an adaptive dehydrated state remain largely unknown.

Although preparation for anhydrobiosis would start with sensing surrounding water evaporation, the mechanisms of sensing desiccation are likely species dependent. Larvae of *P*. *vanderplanki*, can tolerate severe desiccation (less than 5% relative humidity [RH]) after 48 h preconditioning, and surprisingly, brain-deprived larvae also exhibit similar tolerance, suggesting that the central nervous system plays no role in sensing desiccation in this animal [10]. In contrast, dauer larvae of *Caenorhabditis elegans* can tolerate desiccation at 23% RH or above after preconditioning at 98% RH for 4 days [21], and their desiccation tolerance largely depends on two genes, osm11 and osm9, which are expressed in head neurons and required for osmotic avoidance, suggesting that certain head neurons participate in their desiccation tolerance [22]. Therefore, the regulatory mechanisms of desiccation tolerance likely vary among animal species. Tardigrades accumulate only small amounts of trehalose upon desiccation [13], and an anhydrobiotic tardigrade, *Ramazzottius varieornatus*, produces huge amounts of unique heat soluble proteins [19,20], suggesting that tardigrades also have distinct regulatory mechanisms of anhydrobiosis. Almost nothing is known, however, about the anhydrobiotic regulatory mechanisms in tardigrades. *Hypsibius dujardini* is an anhydrobiotic tardigrade which requires longer preconditioning in a high humidity condition to acquire tolerance against severe desiccation [6]. This implies the presence of regulatory mechanisms to induce anhydrobiosis in this species in response to preconditioning. *H*. *dujardini* is easy to maintain in the laboratory, and the strain is established [23] and used for expressed sequence tag and genomic projects, providing plenty of genetic information (http://www.ncbi.nlm.nih.gov/nucest/?term=hypsibius+dujardini). Therefore, this species is suitable for molecular dissection of the regulatory mechanisms of anhydrobiosis in tardigrades.

Here, we used a chemical genetic approach and suggested that *de novo* gene expression is required for entering anhydrobiosis in *H*. *dujardini*. In addition, we identified 6 chemicals that significantly and specifically impaired the anhydrobiotic survival of this tardigrade. Two inhibitors against protein phosphatase (PP) 1 and PP2A exhibited the most profound inhibitory effects, suggesting an important role of PP1/PP2A activity in anhydrobiosis in this species. This is, to our knowledge, the first report of required activities of signaling molecules for desiccation tolerance in tardigrades, and the inhibitory chemicals identified in this study could be powerful tools for further elucidation of the molecular regulatory mechanisms of tardigrade anhydrobiosis.

## Materials and Methods

### Animals

The Z151 strain of *H*. *dujardini* was purchased from Sciento (UK) and maintained at 18°C. Tardigrades were reared on 1.2% agar plates overlaid with volvic water containing *Chlorococcum* sp. (Sciento, UK) as food. Water and food were replaced once or twice a week.

### Chemicals

α-Amanitin, cycloheximide, J-8, and cantharidic acid were purchased from Enzo Life Sciences (USA). Triptolide was purchased from MedChem Express (USA). 3,4-Methylenedioxy-β-nitrostyrene (MNS), 2-aminoethyl diphenylborinate (2-APB), and okadaic acid were purchased from Santa Cruz Biotechnologies (USA). The 81 chemicals used for the screening were provided by the Drug Discovery Initiative, The University of Tokyo (Japan) and are listed in S1 Table. All chemicals were dissolved in dimethyl sulfoxide (DMSO; Wako Pure Chemical, Japan; special grade) as a stock solution and stored at -20°C. Chemical solutions at the appropriate concentrations were prepared by diluting stock solutions in sterilized Milli-Q (stMQ) water just prior to chemical treatment. The final concentration of DMSO in all chemical solutions was adjusted to 1%.

### Desiccation tolerance assay

All procedures were essentially performed at the rearing temperature (18°C). For desiccation, a nylon net filter (Millipore, USA; pore size 11 μm, 25 mm in diameter) was placed on Whatman 3MM filter paper (GE Healthcare, UK; 25 mm diameter) in a plastic dish (35 mm diameter), and tardigrades were dropped onto the net filter with 125 μl of stMQ water. Approximately 20 *H*. *dujardini* were placed on each filter in the experiments other than screening. For preconditioning, the dishes were immediately transferred in a sealed plastic box moistened at a defined RH and incubated for 0 to 4 days. RH was controlled by equilibrium with 60% glycerol (62% RH) or saturated solutions of potassium nitrate (95% RH), potassium chloride (85% RH), or magnesium chloride (33.5% RH) [24,25]. After preconditioning, the animals were exposed to 10% RH in the presence of activated silica gel for 2 days to dehydrate. The dehydrated tardigrades were humidified in 95% RH for 1 day and rehydrated with 2 ml of stMQ water. The number of recovered animals was counted at 1 h and 24 h after rehydration. We defined recovered animals as those exhibiting spontaneous movements or at least responding to touch stimuli. Recovery rates were calculated as the percentage of recovered animals in each population. When necessary, the shapes of the dehydrated tardigrades were observed, and the number of tun-shaped individuals was counted for calculation of the tun formation rates. Animals that were markedly stretched or twisted after desiccation were considered to have non-tun shapes.

### Chemical treatment

The stock solutions were diluted to an appropriate concentration with stMQ water with an adjustment of the DMSO concentration to 1% just prior to chemical treatment. Tardigrades were transferred with 3 μl stMQ water into 50 μl chemical solution of the defined concentration and thus, tardigrades were exposed to slightly diluted concentration of the chemicals (94% of the defined concentration). Tardigrades were incubated in chemical solution for 5 h, except incubation in okadaic acid was for 10 h. Tardigrades in 53 μl solution were then dropped onto a nylon filter placed on the filter paper, and lightly washed with an additional 72 μl stMQ water (total volume: 125 μl) and subjected to desiccation tolerance assays.

### Chemical screening

We screened 81 chemicals that were provided to us by Drug Discovery Initiative, The University of Tokyo (Japan) as a numbered list of compounds. We were blinded to the identification of the compounds. We were not able to analyze all 81 chemicals simultaneously, due to limitation in animal supply and too laborious task. Thus, the 81 chemicals were divided into 20 groups and each group was separately assayed with control treatment (1% DMSO). Ten to 30 *H*. *dujardini* were treated with each chemical solution as described above and subjected to a desiccation series of 95% RH for 2 days, 10% RH for 2 days, and 95% RH for 1 day. Three or four dishes of 10 to 30 tardigrades/dish were assayed for each chemical. The detailed conditions for each group are listed in S1 Table. The recovery rates for each chemical were normalized to the mean recovery rates of tardigrades treated with 1% DMSO (control) in the same group.

### Statistics

Statistical tests were performed using Statcel 3 (OMS, Japan). Specific inhibitory effects on anhydrobiotic survival were examined using the Tukey-Kramer test, comparing four conditions for each chemical. To detect significant inhibition in the chemical screening, the Student’s *t*-test or Dunnett’s test were performed against the DMSO control in the same group.

## Results

### *H*. *dujardini* Z151 strain requires preconditioning to tolerate low humidity exposure

**Fig 1:**
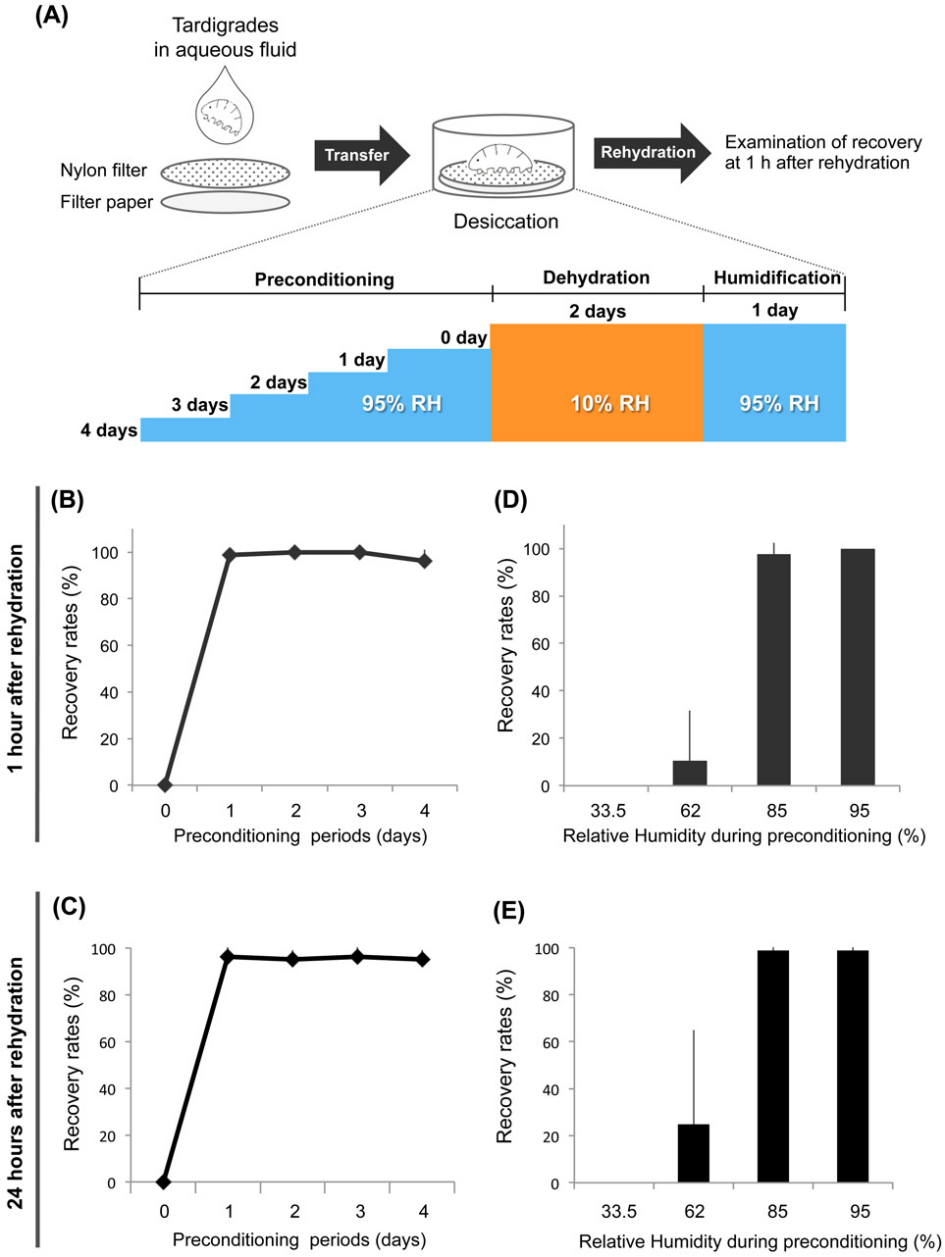
Effects of preconditioning conditions on anhydrobiotic survival in Z151 strain of *H*. *dujardini*. (A) Experimental scheme of the desiccation tolerance assay. Tardigrades in aqueous fluid were dropped onto a nylon filter placed on filter paper, and preconditioned at 95% RH for 0 to 4 days. After preconditioning, they were desiccated at 10% RH for 2 days and humidified at 95% RH for 1 day prior to rehydration. Recovery rates were examined at 1 h after rehydration. (B, C) Effects of various preconditioning periods on anhydrobiotic survival at 1h (B) and 24h (C). Data are shown as mean ± SD (N = 4; 20 tardigrades each). (D, E) Effects of relative humidity exposure during preconditioning on anhydrobiotic survival at 1h (D) and 24h (E). The preconditioning period was fixed as 1 day, and tardigrades were exposed to 33.5%, 62%, 85%, or 95% RH as preconditioning. Data are shown as mean ± SD (N = 4; 20 tardigrades each).

In a previous study, Wright (1989) examined the tolerability of the tardigrade identified as *H*. *dujardini* with an uncertain origin and reported that this species requires pre-incubation at 85% RH for 150 to 200 min to tolerate exposure to a low RH environment (25–31% RH) [6]. The Z151 strain of *H*. *dujardini* was established in 1987 [23] and recently used for various studies, including evo-devo analyses [26] and an expressed sequence tag/genome project, and is thus one of the most suitable tardigrade strains for analyzing the molecular mechanisms activated during preconditioning. Whether the Z151 strain has a similar tolerability as described for *H*. *dujardini* in the previous work, however, is unclear. To address this, we examined the desiccation tolerance of the Z151 strain in a low humidity environment with varying lengths of preconditioning periods (Fig 1A). Without preconditioning, the Z151 strain cannot tolerate exposure to 10% RH (Fig 1B and 1C). A 1-day preconditioning at 95% RH dramatically improved recovery rates to more than 90% and no further differences were detected for longer preconditioning periods of up to 4 days (Fig 1B and 1C). We further examined the effect of varying levels of humidity during the preconditioning period and found that tardigrades preconditioned at 33.5% or 62% RH had no or very low survival after exposure to 10% RH (Fig 1D and 1E). In contrast, tardigrades preconditioned at 85% RH could tolerate 10% RH at levels similar to those at 95% RH (Fig 1D and 1E), though we observed an occasional decrease in the recovery rates when they were preconditioned at 85% RH (data not shown). These findings indicated that the Z151 strain of *H*. *dujardini* requires preconditioning at high humidity, ≥85% RH, to tolerate severe desiccation (10% RH). Preconditioning at 95% RH for 1 day was more reliably sufficient to confer tolerance to exposure to 10% RH for this strain.

### *De novo* gene expression is required for anhydrobiotic survival in *H*. *dujardini*

**Fig 2:**
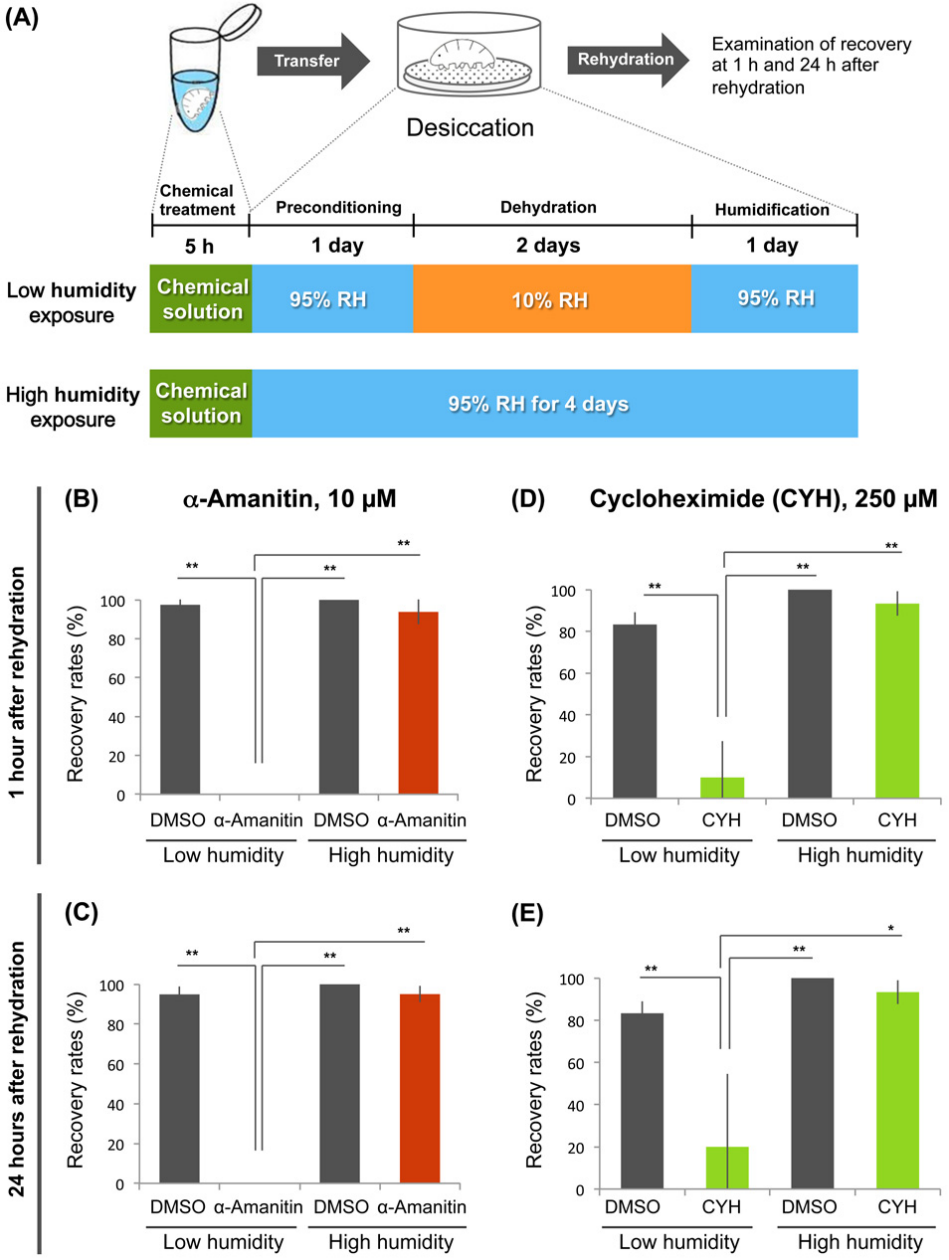
Effects of inhibition of transcription and translation on anhydrobiotic survival. (A) Scheme of chemical treatment and subsequent desiccation tolerance assay. Tardigrades were incubated in a chemical solution or 1% DMSO solution (control) for 5 h. After chemical treatment and 1 day preconditioning at 95% RH, one group was exposed to low relative humidity (10% RH) for 2 days (Low humidity exposure). The other group was exposed to high relative humidity (95% RH) for same period (High humidity exposure). (B, C) Effects of a transcription inhibitor (10 μM α-amanitin) on recovery rates at 1 h (B) and 24 h (C) after rehydration. Mean ± SD (N = 4; 20 tardigrades each). (D, E) Effects of translation inhibitor (250 μM cycloheximide) on recovery rates at 1 h (D) and 24 h (E) after rehydration. Mean ± SD (N = 3; 10 tardigrades each). Statistically significant differences among samples were determined by the Tukey-Kramer test (*, *P*<0.05; **, *P*<0.01). Low humidity, low humidity exposure; High humidity, high humidity exposure.

Many anhydrobiotic animals induce the expression of putative protection molecules upon desiccation, such as late embryogenesis abundant proteins [17,22]. Accordingly, we hypothesized that *H*. *dujardini* also induced the expression of protection molecules necessary for transition to anhydrobiosis during the preconditioning period. To examine this possibility, we pre-treated the tardigrades with the transcription inhibitor α-amanitin [27] for 5 h prior to preconditioning (Fig 2A). Treatment with the transcription inhibitor dramatically reduced the recovery rates from severe desiccation (Fig 2B and 2C). To confirm whether the inhibitor specifically impaired anhydrobiotic survival rather than having general toxic effects, we also examined the effect of the transcription inhibitor on survival after high-humidity treatment, in which exposure to low humidity (10% RH) was replaced with exposure to high humidity (95% RH; Fig 2A). When the tardigrades were exposed to high humidity only, the transcription inhibitor hardly affected their recovery rates, suggesting that α-amanitin specifically impaired anhydrobiotic survival (Fig 2B and 2C). We also examined the effect of the translation inhibitor cycloheximide [28], which had similar results (Fig 2D and 2E). Taken together, these findings suggest that *H*. *dujardini* requires *de novo* transcription and translation for successful transition to anhydrobiosis. The tardigrade likely produces gene products necessary for survival in a low humidity environment during preconditioning periods.

Pre-incubation for 5 h with chemicals was sufficient for both inhibitors against transcription and translation to interfere with anhydrobiotic survival. Thus, we conclude that this experimental scheme is useful for evaluating the effects of various chemicals on anhydrobiotic survival in *H*. *dujardini*.

### Identification of inhibitors of anhydrobiotic survival in *H*. *dujardini*

**Fig 3:**
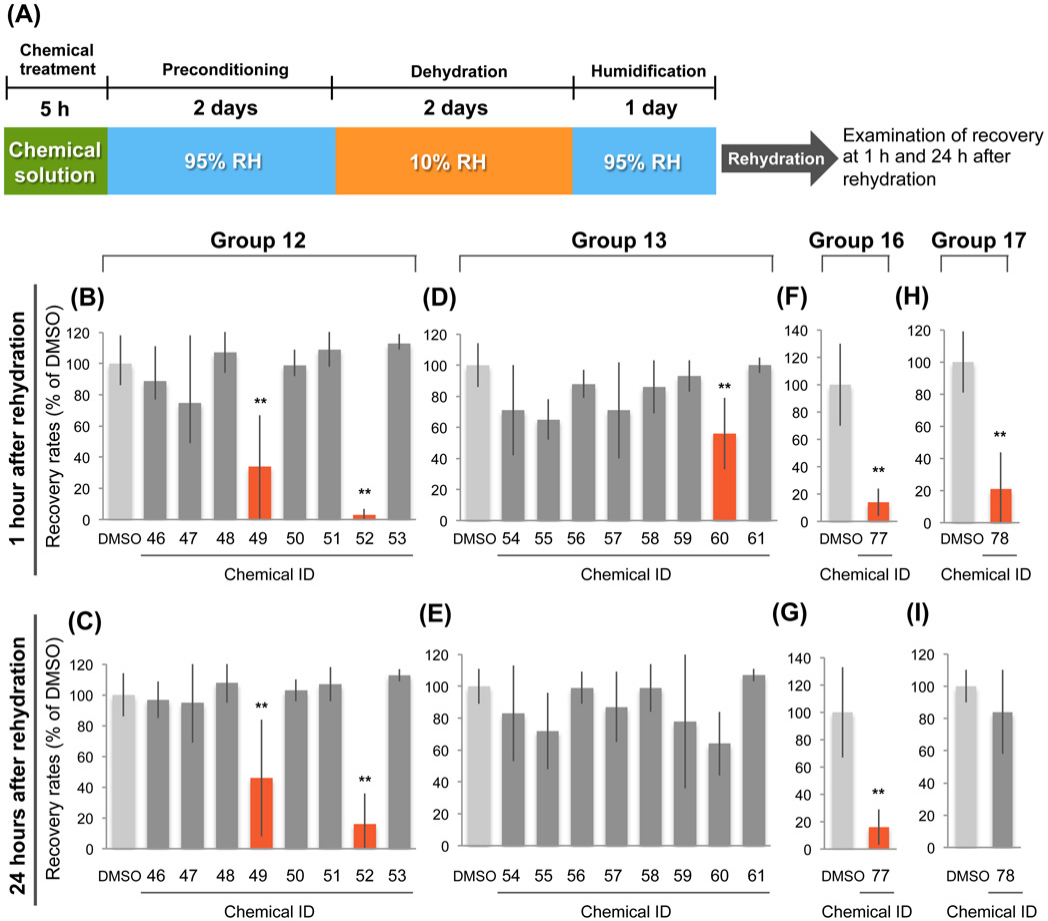
Screening of chemicals inhibiting anhydrobiotic survival. (A) Experimental scheme used for chemical screening. (B-I) The effects of various chemicals on anhydrobiotic survival in the four experimental groups that contained the chemicals that significantly inhibited anhydrobiotic survival. Significant inhibitory effects were detected for chemicals #49 (J-8) and #52 (triptolide) of Group 12 (B, C); chemical #60 (MNS) of Group 13 (D); chemical #77 (cantharidic acid) of Group 16 (F, G); and chemical #78 (2-APB) of Group 17 (H). At 24 h after rehydration, no significant inhibitory effects of chemical #60 of Group 13 and chemical #78 of Group 17 were detected (E, I). Recovery rates are shown as percent of DMSO control. Mean ± SD (N = 4; 30 tardigrades each). In each group, statistically significant differences from the DMSO control were determined by Dunnett’s test (Groups 12 and 13) or Student’s *t*-test (Groups 16 and 17), **, *P*<0.01.

The requirement of *de novo* gene expression for anhydrobiotic survival prompted us to postulate the presence of signal transduction pathways connecting desiccation stimuli to responsive expression of genes related to desiccation tolerance. To elucidate such signaling pathways, we conducted chemical screening to identify chemicals that inhibit anhydrobiotic survival in *H*. *dujardini*. We selected 81 chemicals as candidates from available inhibitory chemicals whose target molecules were known, based on knowledge of the stress-responsive pathways in other organisms, general signaling molecules, and the gene repertoire encoded in the tardigrade genome (S1 Table). Chemical treatment was performed mostly at predefined concentrations such as 100 μM or 20 μM, depending on the solubility of the chemicals in DMSO. When the animals appeared immotile after chemical treatment, the concentration was decreased to avoid immobility of the animals. After screening the 81 chemicals, we identified 5 chemicals that significantly inhibited anhydrobiotic survival (Fig 3 and S1 Table). These five chemicals included a calmodulin antagonist (J-8) [29], an immunosuppressive agent (triptolide) [30], a Syk and Src kinase inhibitor (MNS) [31], a PP1 and PP2A inhibitor (cantharidic acid) [32], and a calcium release modulator (2-APB) [33]. Triptolide and cantharidic acid exhibited much stronger inhibitory effects than the other three chemicals at 1 h as well as 24 h after rehydration (Fig 3B, 3C, 3F and 3G), suggesting that their target molecules play an important role in the transition to anhydrobiosis. For MNS and 2-APB, significant inhibition of the recovery rates was detected only at 1 h after rehydration (Fig 3D, 3E, 3H and 3I).

### Identified chemicals specifically impaired anhydrobiotic survival in *H*. *dujardini*

**Fig 4:**
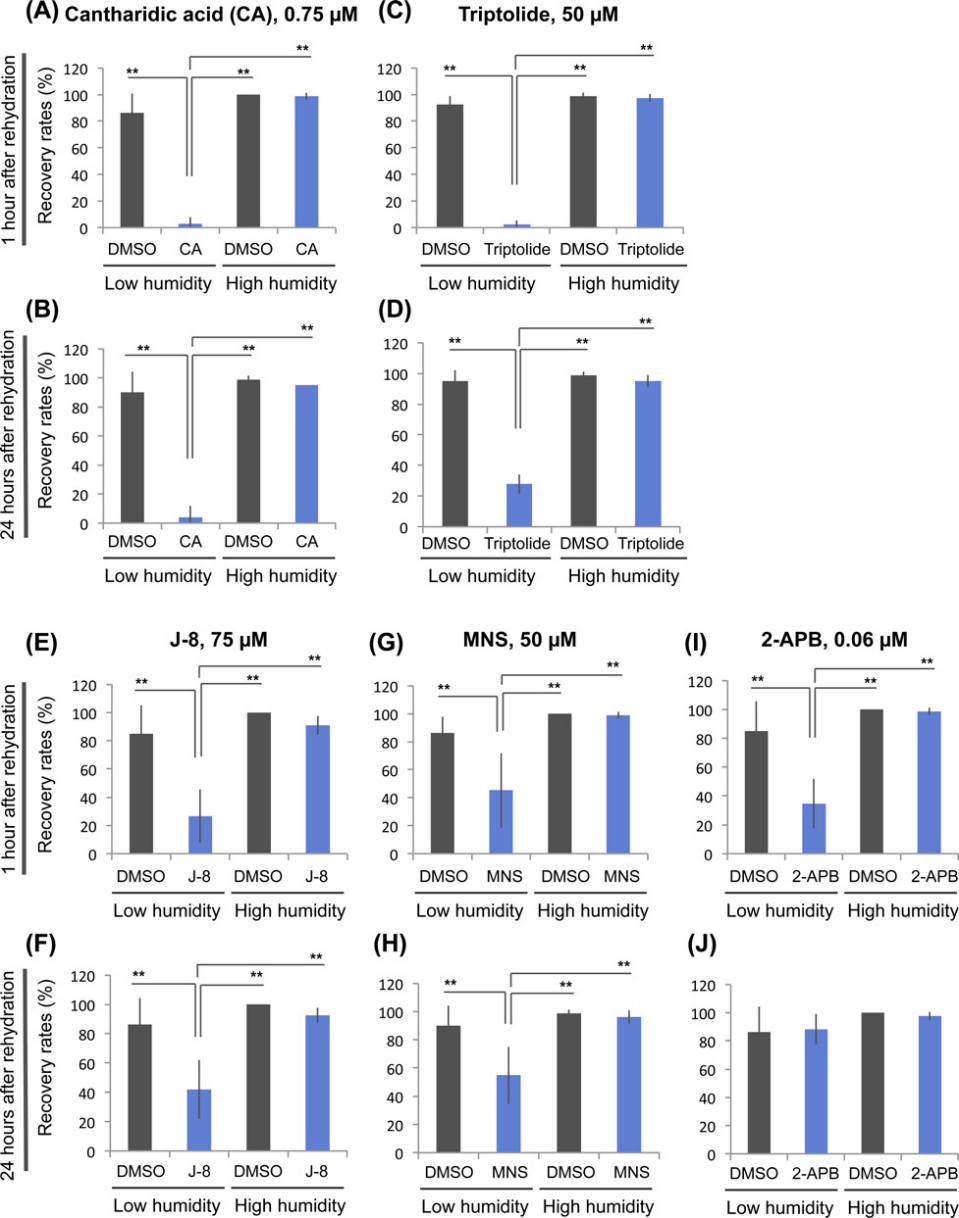
Specific inhibition of anhydrobiotic survival by identified chemicals. The specificity of the inhibitory effects on anhydrobiotic survival was examined for the five identified chemicals using essentially the same experimental scheme shown in Fig 2A. Effects on recovery rates are shown for 0.75 μM cantharidic acid (CA) (A, B); 50 μM triptolide (C, D); 75 μM J-8 (E, F); 50 μM MNS (G, H); and 0.06 μM 2-APB (I, J). Recovery rates were examined at both 1 h (A, C, E, G, I) and 24 h (B, D, F, H, J) after rehydration. Mean ± SD (N = 4; 20 tardigrades each). Statistically significant differences among samples were determined by the Tukey-Kramer test (**, *P*<0.01). Low humidity, low humidity exposure; High humidity, high humidity exposure.

To elucidate whether the identified chemicals specifically impaired anhydrobiotic survival rather than having nonspecific toxicity, we examined the effects of the chemicals on survival of tardigrades exposed only to high humidity (95% RH) instead of exposure to low humidity (10% RH). At the appropriate concentrations, all five chemicals exhibited significant inhibitory effects specifically on survival after exposure to low humidity, with little effect on survival after exposure to only high humidity (Fig 4 and S1 Fig). These findings suggest that all five identified chemicals have specific inhibitory effects on anhydrobiotic survival.

### Inhibition of PP1/PP2A activity impaired anhydrobiotic survival in *H*. *dujardini*

**Fig 5:**
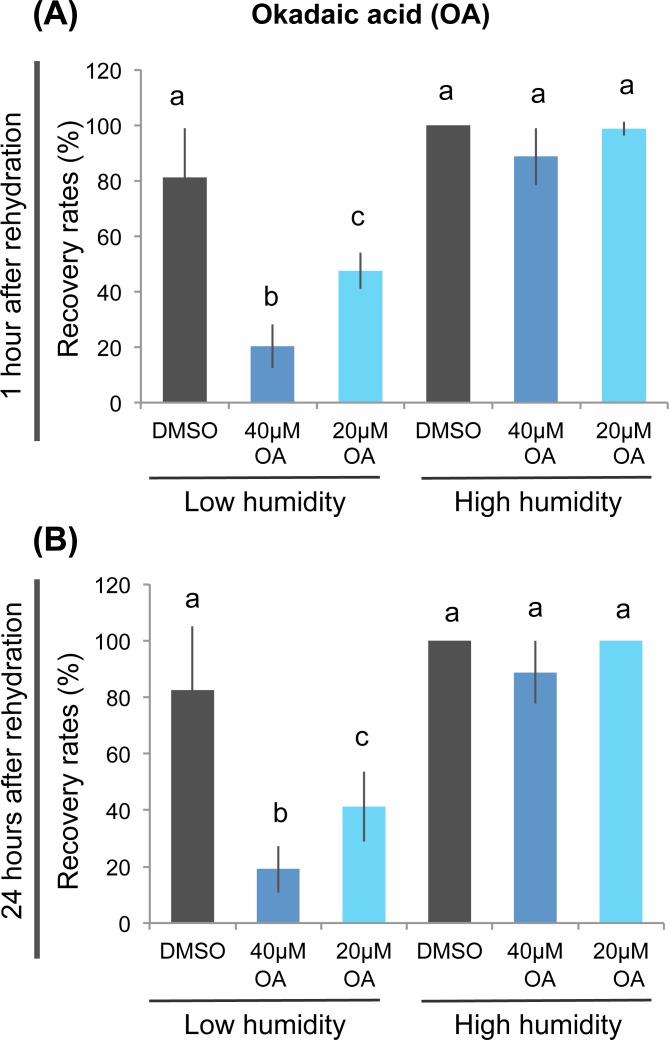
Inhibition of PP1/PP2A activity impaired anhydrobiotic survival. The effects of another potent PP1/PP2A inhibitor, okadaic acid, on anhydrobiotic survival were examined using essentially the same scheme shown in Fig 2A, with a longer chemical treatment period (10 h). Either 40 μM or 20 μM okadaic acid specifically inhibited recovery rates after low humidity exposure at both 1 h (A) and 24 h (B) after rehydration. Mean ± SD (N = 4; 20 tardigrades each). Statistically significant differences among samples were determined by the Tukey-Kramer test. Different letters indicate significant differences (*P*<0.01). Low humidity, low humidity exposure; High humidity, high humidity exposure.

Among the five identified chemicals, cantharidic acid and triptolide exhibited the most profound inhibitory effects on anhydrobiotic survival (Fig 4A–4D and S1A–S1D Fig). Cantharidic acid is a highly selective inhibitor against PP1 and PP2A [32], while triptolide interacts with various biologic molecules and thus it is difficult to determine the important target for anhydrobiosis [34–37]. Therefore, we focused on PP1/PP2A, the target of cantharidic acid. To confirm whether PP1/PP2A activity is required for anhydrobiosis, we examined the effect of another potent PP1/PP2A inhibitor, okadaic acid [38]. Okadaic acid also significantly inhibited recovery from severe desiccation, whereas no toxic effects were observed when the tardigrades were exposed to high humidity only, suggesting that okadaic acid also specifically impaired anhydrobiotic survival (Fig 5). Taken together with the effect of cantharidic acid, these findings suggest that PP1/PP2A activity plays an important role in anhydrobiotic survival in *H*. *dujardini*.

## Discussion

The findings of the present study indicated that inhibiting either transcription or translation severely impaired anhydrobiotic survival of *H*. *dujardini* (Fig 2). To our knowledge, this is the first report to clarify the requirement of *de novo* gene expression for successful transition to anhydrobiosis in tardigrades. In addition, we identified six additional chemicals that specifically inhibited survival from severe desiccation (Figs 3–5 and S1 Table), and two inhibitors against PP1/PP2A exhibited particularly profound inhibitory effects, suggesting an important role of PP1/PP2A activity in anhydrobiosis of tardigrades.

Requirement of *de novo* gene expression suggests that tardigrades sense mild desiccation during preconditioning and synthesize new transcripts to prepare the necessary molecules to tolerate severe dehydration. This implies that newly synthesized transcripts during preconditioning are candidate molecules involved in the transition to anhydrobiosis, and thus, based on this evidence, comparative analyses of gene expression during preconditioning appears to be a promising strategy for identifying anhydrobiosis-related genes using this tardigrade. In the present study, we used α-amanitin, a selective inhibitor of RNA polymerase II [27], and thus synthesized messenger RNA is a primary candidate molecule involved in anhydrobiosis. This does not rule out the possible roles of other RNA molecules transcribed by other polymerases, such as some non-coding RNAs transcribed by RNA polymerase III [39]. Chemical inhibition of RNA polymerase III, however, also affects the synthesis of transfer RNA, making it difficult to clarify the role of such non-coding RNA in anhydrobiosis because inhibition of translation itself affected anhyrobiotic survival (Fig 2D and 2E). Therefore, total RNA-seq analyses would be the most suitable strategy for identifying differentially expressed genes, including noncoding RNA.

In addition to transcriptional regulation, our chemical screening suggested that multiple signaling pathways are also required for successful anhydrobiosis, possibly in the signal transduction of desiccation stimuli to appropriate gene expression. Especially, dephosphorylation by PP1/PP2A could play an important role in acquiring tolerance against severe desiccation in *H*. *dujardini*, because two independent PP1/PP2A inhibitors, cantharidic acid and okadaic acid, exhibited profound inhibitory effects on anhydrobiotic survival (Figs 4 and 5 and S1 Fig). Although these inhibitors suppressed survival only when animals were exposed to low humidity condition, suggesting their specific inhibitory effects, we could not exclude the possibility that nonspecific toxic action of these inhibitors might reach lethality only when animals were exposed to severe desiccation in low humidity condition. This is limitation of chemical approaches and other loss of function experiments, such as knock down by RNAi, will consolidate the requirement of corresponding signaling molecules, including PP1/PP2A. PP1/PP2A are multimeric serine/threonine protein phosphatases composed of a highly conserved catalytic subunit and various regulatory subunits, and PP2A also contains a scaffolding subunit [40,41]. Cantharidic acid and okadaic acid inhibit both catalytic subunits of PP1 and PP2A [40]. Regulatory subunits determine the substrate specificity of PP1/PP2A holoenzymes and are thus involved in specific biologic processes. In budding yeast, *Saccharomyces cerevisiae*, one regulatory subunit of PP2A, Cdc55, is required for full activation of osmotic stress response genes via indirect regulation of stress-responsive transcription factors Msn2/Msn4 [42]. Because mild dehydration during preconditioning likely causes osmotic stress, tardigrades might possess a similar pathway contributing to anhydrobiosis. In various animals, PP2A is involved in the positive regulation of forkhead transcription factor, FOXO, via inactivation of Akt kinase or direct dephosphorylation [43–47]. FOXO is an important transcription factor involved in longevity, stress-resistance, and regulation of metabolism downstream of insulin/insulin-like growth factor signaling [48]. Especially, FOXO upregulates the transcription of antioxidant enzymes such as manganese superoxide dismutase, and increases resistance to oxidative stress [49,50]. Desiccation stress enhances the accumulation of reactive oxygen species [51,52], and thus FOXO activation through PP2A might be involved in the anhydrobiotic response during preconditioning in tardigrades.

In addition to PP2A, PP1 activity may also be involved in anhydrobiosis. In dauer larvae of *C*. *elegans*, myosin light chain (MLC) is clearly dephosphorylated during the preconditioning period [22], although its significance in desiccation tolerance remains elusive. Dephosphorylation of MLC is mostly catalyzed by MLC phosphatase, whose catalytic subunit is the same as that of PP1, the target of cantharidic acid and okadaic acid [40]. Thus, dephosphorylation by PP1 could be a common process during preconditioning in *H*. *dujardini* and dauer larvae of *C*. *elegans*.

In addition to phosphatase inhibitors, MNS, a protein tyrosine kinase inhibitor, also significantly inhibited anhydrobiotic survival (Fig 4G and 4H and S1G and S1H Fig). MNS is a selective inhibitor of Syk and Src kinases [31]. In vertebrates, Syk and Src are involved in the response to oxidative stress [53–55], and Syk is also involved in the response to osmotic stress in chicken B cells [56]. As described above, oxidative and osmotic stresses likely occur during desiccation stress; thus, Syk and Src might be involved in mitigation of these stresses during preconditioning in tardigrades. Their contributions to anhydrobiosis, however, might only be partial, because the inhibitory effect of MNS was rather moderate compared to that of cantharidic acid (Fig 4 and S1 Fig).

Mitogen-activated protein kinase (MAPK) pathways are representative phosphorylation signaling pathways involved in various environmental stress responses [57,58]. These pathways are highly conserved in eukaryotic organisms and are well-studied. In an anhydrobiotic nematode, *Panagrolaimus superbus*, pre-treatment with MAPK inhibitors against JNK (SP600125; 50 μM or 100 μM) and p38 (SB239063; 50 μM or 100 μM) partially inhibits anhydrobiotic survival only when nematodes were exposed to 10% RH without preconditioning [59]. In the same report, however, anhydrobiotic survival after preconditioning was almost unaffected by these MAPK inhibitors [59]. Correspondingly, we detected no significant effects of the same MAPK inhibitors, such as SP600125 (JNK; Chemical ID 1) and SB239063 (p38; Chemical ID 50), and also no significant effect by U0126 (MEK1/MEK2; Chemical ID 47) [60] at the same concentration of 100 μM of these inhibitors (S1 Table). Thus, MAPK signaling is likely not essential for anhydrobiosis in preconditioned tardigrades as well as preconditioned nematodes. We cannot, however, exclude the possibility that in the tardigrade, these chemicals could not effectively penetrate or inhibit the target kinases or concentrations used in this study were not appropriate.

Triptolide was another chemical with strong inhibitory effects (Fig 4C and 4D and S1C and S1D Fig). This compound was originally identified as an immunosuppressive agent. Triptolide indirectly interferes with the NF-κB pathway and suppresses immune responses [30]. After its discovery, many other target molecules were identified for triptolide, e.g., calcium channel polycystin-2 [34–37]. One of the target proteins is XPB, a subunit of the core transcription factor TF-II H, and triptolide also inhibits general transcription [37,61]. Therefore, the strong suppression of triptolide on anhydrobiotic survival could be due to its inhibitory effect on transcription, which is consistent with the effects of α-amanitin (Fig 2B and 2C). Inhibitory effects on other targets could also contribute to suppression of anhydrobiotic survival.

The other two identified inhibitors, J-8 and 2-APB, are a calmodulin antagonist and calcium release modulator, respectively [29,33]. These findings suggest that calcium signaling is involved in anhydrobiotic survival in *H*. *dujardini*, in part because their inhibitory effects were relatively weak (Fig 4 and S1 Fig). Calcium is a general signaling molecule that regulates the activities of various enzymes, mostly through binding with a partner protein, calmodulin (CaM). Recent work revealed that CaM regulates c-Src kinase activity, and the CaM antagonist W-7 inhibits the activation of c-Src in response to hydrogen peroxide-induced oxidative stress [62]. As described above, the Src inhibitor MNS also inhibited anhydrobiotic survival at a level comparable to that of the CaM antagonist J-8 (Fig 4 and S1 Fig). Calcium signaling and c-Src might play a role in the same pathway in anhydrobiosis in the tardigrade. We could not detect significant effects of various inhibitors against other CaM-regulated enzymes (S1 Table), such as CaMK2 (Chemical ID 15) [63], calcineurin (Chemical ID 29) [64], nitric oxide synthase (Chemical ID 55) [65], phosphodiesterase type 1 (Chemical ID 43) [66], and MLC kinase (Chemical ID 80) [67], nor inhibitors against other calcium-related proteins, like calcium channels (e.g., Chemical ID 5) [68], possibly due to their small contribution to anhydrobiosis or to insufficient inhibition of target proteins in tardigrades.

In summary, the findings of the present study suggested the requirement of *de novo* gene expression in anhydrobiosis, and provide evidence that comparative transcriptome analyses are a promising strategy for identifying anhydrobiosis-related genes. The newly identified six inhibitory chemicals, including two PP1/PP2A inhibitors, could become powerful tools for the molecular dissection of the regulatory mechanisms of anhydrobiosis in tardigrades.

## Supporting Information

S1 Fig Specific inhibition of anhydrobiotic survival by identified chemicals. Specific inhibitory effects on anhydrobiotic survival were reproduced for all five identified chemicals. The optimal concentration varied slightly for J-8 and 2-APB (see Fig 4), possibly due to the physiologic condition of the cultured tardigrades. Effects on recovery rates are shown as mean ± SD for 0.75 μM cantharidic acid (CA) (A, B); 50 μM triptolide (C, D); 50 μM J-8 (E, F); 50 μM MNS (G, H); and 0.02 μM 2-APB (I, J). Recovery rates were examined at both 1 h (A, C, E, G, I) and 24 h (B, D, F, H, J) after rehydration. N = 4 unless otherwise stated; 20 tardigrades each. Statistically significant differences among samples were determined by the Tukey-Kramer test (*, *P*<0.05; **, *P*<0.01). Low humidity, low humidity exposure; High humidity, high humidity exposure.
(TIF)

S2 Fig Variable effects of identified chemicals on tun formation. The effects of identified chemicals on tun formation were examined. The effects on tun formation are generally much smaller than those on anhydrobiotic survival, and the significance of the effects varied among experiments, suggesting that these chemicals have subtle inhibitory effects on tun formation, if any. Tun formation rates are shown for experiments corresponding to Fig 4A, 4C, 4E, 4G and 4I and S1B, S1D, S1F, S1H, S1J Fig). (A, B) cantharidic acid (CA), (C, D) triptolide, (E, F) J-8, (G, H) MNS, and (I, J) 2-APB. N = 4 unless otherwise stated; 20 tardigrades each. Statistically significant differences among samples were determined by the Tukey-Kramer test (*, *P*<0.05; **, *P*<0.01). Low humidity, low humidity exposure; High humidity, high humidity exposure.
(TIF)

S1 Table List of chemicals assayed in the screening and results. The effects of 81 chemicals on anhydrobiotic survival were examined. The 81 chemicals were divided into 20 groups and each group was assayed independently with a separate DMSO-treated control for each group. Tardigrades were treated with chemical solution or 1% DMSO solution (control; blue highlighting) for 5 h prior to the desiccation tolerance assay. After chemical treatment, they were preconditioned at 95% RH for 2 days, followed by dehydration at 10% RH for 2 days, and humidification at 95% RH for 1 day. Recovery was examined at 1 h and 24 h after rehydration. In the list, recovery rates are shown as raw data as well as relative values normalized with that of DMSO-treated control (% of DMSO) in the same group (N = 3 or 4; 10 to 30 tardigrades each). For each group, statistically significant differences compared with the DMSO-treated control were determined using Dunnett’s test (Groups 1–15) or Student’s *t*-test (Groups 16–20). Pink highlighting indicates five chemicals that exhibited significant inhibitory effects (*P*<0.01). With regard to the other chemicals that did not exhibit significant inhibitory effects, we cannot exclude the possibility that concentrations used in the screening were not appropriate and/or had insufficient effects on the tardigrade.
(XLS)
