# Supplementary figures and images for: Suggested Involvement of PP1/PP2A Activity and De Novo Gene Expression in Anhydrobiotic Survival in a Tardigrade, Hypsibius dujardini, by Chemical Genetic Approach
Source: PLoS One. 2015 Dec 21;10(12):e0144803. doi: 10.1371/journal.pone.0144803 (PMC4686906; doi:10.1371/journal.pone.0144803)

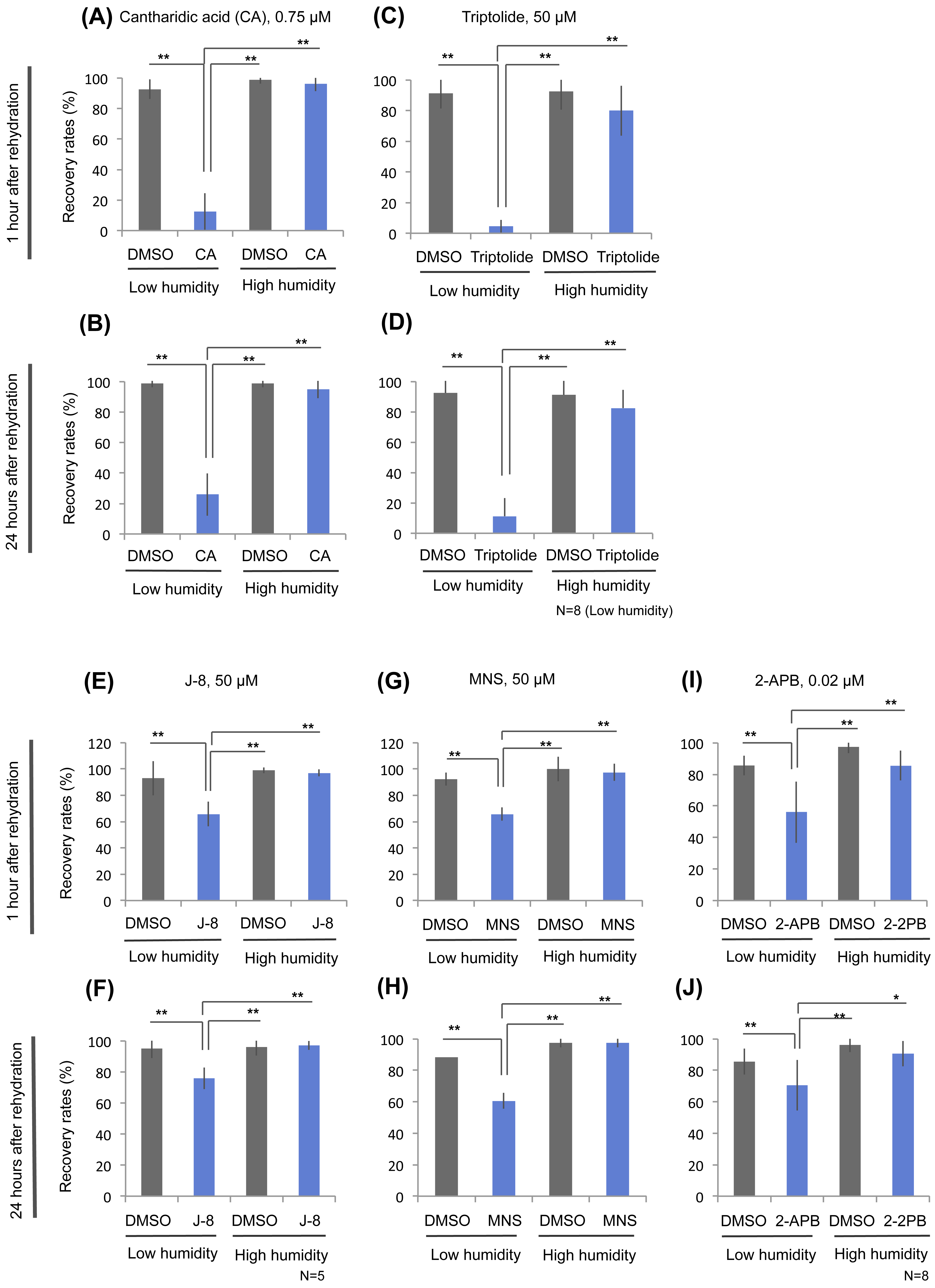

Supplement: S1 Fig — Specific inhibitory effects on anhydrobiotic survival were reproduced for all five identified chemicals. The optimal concentration varied slightly for J-8 and 2-APB (see Fig 4), possibly due to the physiologic condition of the cultured tardigrades. Effects on recovery rates are shown as mean ± SD for 0.75 μM cantharidic acid (CA) (A, B); 50 μM triptolide (C, D); 50 μM J-8 (E, F); 50 μM MNS (G, H); and 0.02 μM 2-APB (I, J). Recovery rates were examined at both 1 h (A, C, E, G, I) and 24 h (B, D, F, H, J) after rehydration. N = 4 unless otherwise stated; 20 tardigrades each. Statistically significant differences among samples were determined by the Tukey-Kramer test (*, P<0.05; **, P<0.01). Low humidity, low humidity exposure; High humidity, high humidity exposure. (TIF) [file pone.0144803.s001.tif]

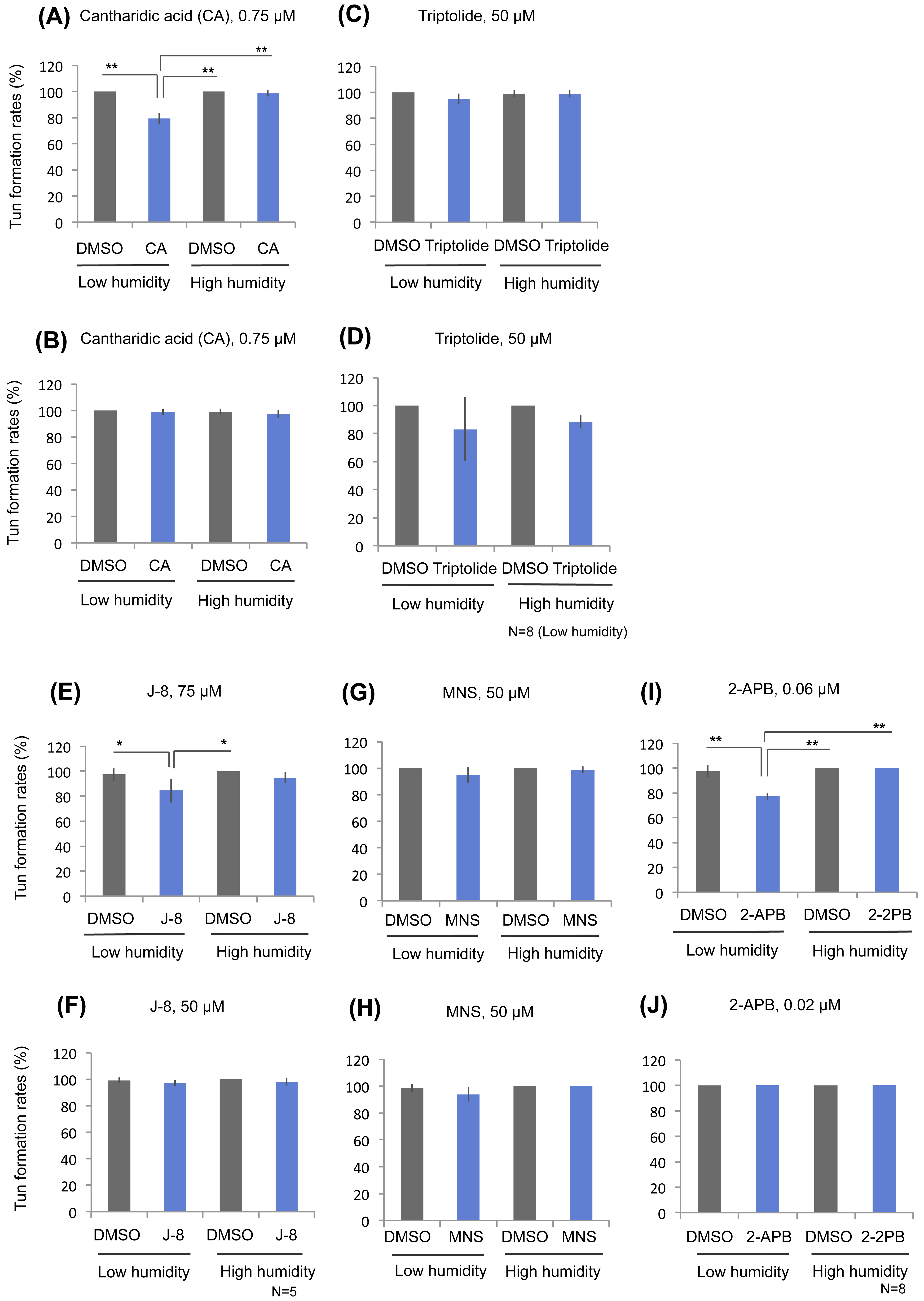

Supplement: S2 Fig — The effects of identified chemicals on tun formation were examined. The effects on tun formation are generally much smaller than those on anhydrobiotic survival, and the significance of the effects varied among experiments, suggesting that these chemicals have subtle inhibitory effects on tun formation, if any. Tun formation rates are shown for experiments corresponding to Fig 4A, 4C, 4E, 4G and 4I and S1B, S1D, S1F, S1H, S1J Fig). (A, B) cantharidic acid (CA), (C, D) triptolide, (E, F) J-8, (G, H) MNS, and (I, J) 2-APB. N = 4 unless otherwise stated; 20 tardigrades each. Statistically significant differences among samples were determined by the Tukey-Kramer test (*, P<0.05; **, P<0.01). Low humidity, low humidity exposure; High humidity, high humidity exposure. (TIF) [file pone.0144803.s002.tif]
